# Anthropometric failures and its predictors among under five children in Ethiopia: multilevel logistic regression model using 2019 Ethiopian demographic and health survey data

**DOI:** 10.1186/s12889-024-18625-4

**Published:** 2024-04-24

**Authors:** Aznamariam Ayres, Yeshimebet Ali Dawed, Shambel Wedajo, Tilahun Dessie Alene, Alemu Gedefie, Fekadeselassie Belege Getahun, Amare Muche

**Affiliations:** 1 Department of Epidemiology and Biostatistics, School of Public Health, College of Medicine and Health Sciences, Wollo University, Dessie, Ethiopia; 2 Department of Nutrition, School of Public Health, College of Medicine and Health Sciences, Wollo University, Dessie, Ethiopia; 3 School of Public Health, CMHS, Wollo University, Dessie, Ethiopia; 4 Department of Paediatrics, School of Medicine, College of Medicine and Health Sciences, Wollo University, Dessie, Ethiopia; 5 Department of Medical Laboratory Sciences, College of Medicine and Health Sciences, Wollo University, Dessie, Ethiopia; 6 Department of Paediatrics Neonatal & Child Health, School of Nursing and Midwifery, College of Medicine and Health Sciences, Wollo University, Dessie, Ethiopia

**Keywords:** Composite index of anthropometric failure, Under five children, Ethiopia

## Abstract

**Background:**

Composite Index of Anthropometric Failure (CIAF) combines all three forms of anthropometric failures to assess undernutrition status of children. There is no study on CIAF to identify the real and severe form of under nutrition among Ethiopian children that addressed community level factors. So, this study determined CIAF and identified important factors which helps to design appropriate intervention strategies by using multi-level advanced statistical model.

**Methods:**

The study included 5,530 under five children and utilized a secondary data (EMDHS 2019) which was collected through community-based and cross-sectionally from March 21 to June 28, 2019. Composite index of anthropometric failure among under five children was assessed and a two-stage sampling technique was used to select the study participants. Descriptive summary statistics was computed. A multi-level binary logistic regression model was employed to identify important predictors of CIAF in under five children. Adjusted odds ratio with its 95% CI was estimated and level of significance 0.05 was used to determine significant predictors of CIAF.

**Results:**

The prevalence of composite index of anthropometric failure (CIAF) was 40.69% (95% CI: 39.41, 42.00) in Ethiopia. Both individual and community level predictors were identified for CIAF in under five children. Among individual level predictors being male sex, older age, short birth interval, from mothers who have not formal education, and from poor household wealth quintile were associated with higher odds of CIAF among under five children. Low community women literacy and being from agriculturally based regions were the community level predictors that were associated with higher odds of CIAF in under five children in Ethiopia.

**Conclusions:**

The burden of composite index of anthropometric failure in under five children was high in Ethiopia. Age of child, sex of child, preceding birth interval, mother’s education, household wealth index, community women literacy and administrative regions of Ethiopia were identified as significant predictors of CIAF. Therefore, emphasis should be given for those factors to decrease the prevalence of CIAF in under five children in Ethiopia.

## Background

Under-nutrition is a major public health concern in many developing nations, including Ethiopia, and is a global concern. Additionally, it is still one of the main causes of disease, early mortality, and morbidity in these countries' children [1–3].

Under-nutrition in the population of under-five children is commonly measured by three anthropometric indicators, namely stunting (low height for age), wasting (low weight for height), and underweight (low weight for age). Stunting and wasting are symptoms of chronic and acute dietary deficits, respectively. Additionally, both acute and chronic dietary deficiencies are reflected by underweight [4, 5]. This approach is used to evaluate the size, proportion and composition of the human body. Children's general health status, dietary sufficiency, and growth and development patterns can all be assessed using anthropometry [6, 7].

In order to address the issue of multiple nutritional failures and to report the prevalence of real data, the Composite Index of Anthropometric Failure (CIAF) method was developed. This method can be used to identify children who have one or more anthropometric failure(s) [8]. The CIAF is an anthropometric index that combines the three indices of weight-for-age, height/length-for-age, and weight-for height/length in order to assess the nutritional status of children under five years of age [9]. The combined index method of the Svedberg model creates six categories of undernourished children: A) without anthropometric failure; B) wasting only; C) wasting and underweight; D) wasting, underweight, and stunting; E) underweight and stunting; F) stunting only. Additionally, Nandy et al. added category Y), which is underweight only [10].

In 2019, globally there were 144 million stunted, 47 million wasted, and 38 million over weighted children under the age of five [11]. According to a World Bank report on under-five child malnutrition, Asian children account for 56% of stunted children, while African children for 37% of stunted children and 25% of wasted children [12]. Results from the Global Burden of Diseases, Injuries, and Risk Factors Study 2016 (GBD 2016) indicate that in Sub-Saharan Africa (SSA), an estimated 36.6% of children under five were stunted, 8.6% were wasting, and 19.5% were underweight in 2015 [13]. In Ethiopia according to EMDHS 2019 report, there were 37% stunted children under five, 7% wasted children, and 21% underweight children [14].

The majority of earlier research on undernutrition prevalence and contributing factors in Ethiopia has concentrated on a particular undernutrition indicator, such as stunting, wasting, and underweight [8, 15–19], separately proposed by the World Health Organization (WHO) [20]. But because the children in those conventional indices overlapped into numerous categories of anthropometric failure, they severely underestimated the prevalence and were unable to provide accurate estimates of the true cost of childhood undernutrition. This is due to the fact that the commonly used indices may overlap, making it possible for a single kid to exhibit signs of two or more undernutrition indicators at the same time. As a result, the indices may not be sufficient to accurately assess the true burden of undernutrition situations among children under five [3, 8, 16, 17, 19, 21–26]. By combining the common indicators of undernutrition measures, the creation of the composite index of anthropometric failure (CIAF) gets beyond these restrictions [8, 15–17, 27]. Literatures support the use of CIAF rather than using traditional (conventional) methods for the assessment of child undernutrion status [28, 29].

This study provides new estimates for the prevalence of undernutrition by aggregating traditional undernutrition indices which is important to capture the overall impact of under nutrition on a population unlike that of any of the three traditional indicators. Even if there was a limited study conducted regarding childhood under-nutrition using CIAF in Ethiopia which was a small scale surveys [30] limited in particular regions of the country. The current study was incorporates variables like region, residence, community women education level, and poverty level to determine how they affect CIAF. Therefore, to address the above identified gaps this study aim to assess undernutrition using the CIAF and its predictor on children under the age of five in Ethiopia using multi-level advanced statistical model.

## Methods

### Study design and setting

Community-based cross-sectional study design was employed among under five children in Ethiopia. Administratively, Ethiopia is divided into nine geographical regions and two administrative cities. Ethiopia is located in the horn of Africa covering 1,104, 300 km^2^ that ranks 10th in Africa in land coverage.

So far, in Ethiopia about six Demographic and Health Surveys (four main and two mini) conducted in Ethiopia. The 2019 EMDHS is the latest survey conducted in Ethiopia. The Ethiopian Public Health Institute (EPHI) conducted the survey at the need of Ethiopian Federal Ministry of Health (EFMoH). Financial support was provided by the government of Ethiopia, World Bank through the Ministry of Finance and Economic Development’s Enhancing shared Prosperity through Equitable Services (ESPES) and Promoting Basic Services (PBS) projects, the United Nations Children’s Fund (UNICEF), and the United States agency for International Development (USAID). Inner City Fund (ICF) provided technical support via the DHS program, which is funded by the USAID and offers support and technical assistance for the implementation of population and health surveys in countries worldwide. The 2019 Ethiopian Mini Demographic and Health Survey (EMDHS 2019) is the second Mini Demographic and Health Survey and the sixth Demographic and Health Survey conducted in Ethiopia, which was conducted from March 21, 2019 to June 28, 2019 [14].

### Population

The source population is under five children in Ethiopia in EMDHS 2019 survey time. The study population is under five children who were living in the selected enumeration areas during EMDHS 2019 in Ethiopia. All under five children in Ethiopia were included to this study. All under five children who fulfill the inclusion criteria but children who had incomplete data [14].

### Sample size and sampling procedures

Since this study used a secondary data and the study participants were 5,530 under five children. The data set is large and collected nationwide across all regions of Ethiopia. The sampling frame used for the 2019 EMDHS was a frame of all census enumeration areas (EAs) created for the 2019 Ethiopia Population and Housing Census (EPHC) and conducted by the Central Statistical Agency (CSA). The census frame was a complete list of the 149,093 EAs created for the 2019 EPHC. An EA is a geographic area covering an average of 131 households. The data from a DHS survey naturally forms a hierarchy of household within a cluster, household members within each household, interviewed women and men as a subset of household members, and children of each interviewed woman [14].

The 2019 EDHS sample was stratified and selected in two stages. Each region was stratified in to urban and rural areas, yielding 21 sampling strata. Samples of EAs were selected independently in each stratum in two stages. Implicit stratification and proportional allocation were achieved at each of the lower administrative levels by sorting the sampling frame within each sampling stratum before sample selection, according to administrative units in different units in different levels [14].

In the first stage, a total of 305 EAs (93 from urban and 212 from rural areas) were selected with probability proportional to EA size and with independent selection in each sampling stratum. A household listing operation was carried out in all selected EAs from January through April 2019. The resulting lists of households served as a sampling frame for the selection of households in the second stage. In the second stage of selection, a fixed number of 30 households per cluster were selected with an equal probability of systematic selection from the newly created household listing, and all under five children who were either permanent residents or visitors and slept in the household the night before the survey were eligible for an interview [14, 31].

### Variables of the study

The theoretical framework how the individual and the community-level factors contribute to the Campsite Index of Anthropometric Failure (CIAF) supported by the ecological model that individual development is influenced by nested layers of the environment, from individual characteristics (like age) to broader societal factors (like socioeconomic status).

Composite Index of Anthropometric Failure (CIAF) is not solely determined by individual choices and behaviors. Social, economic, and environmental factors within communities significantly influence CIAF outcomes. By incorporating these community-level variables, we can move beyond individual-level explanations and uncover broader societal influences on CIAF.

#### Dependent variable

Children composite index of anthropometric failure coded as 1 = yes, 0 = no.

#### Stratifier variable

Cluster number (enumeration area) was the stratifier variable (v001 which ranges from 1 to 305 enumeration areas).

### Independent variables

#### Level 1 factors: Individual level and household level characteristics

Mother’s education, Mother’s age, Mother’s BMI, child age, child sex, birth type, birth order, birth interval, mothers’ education, Wealth index, drinking water source, type of toilet facility.

#### Level 2 factors: Community level characteristics

Region, residence, community poverty level, community women literacy, community mass media exposure.

## Operational definitions

*Composite index of anthropometric failure (CIAF)*: is an anthropometric index that combines weight-for-age (WAZ), length/height-for-age (HAZ) and weight-for-length (WHZ) to determine the nutritional status of children under five. The category of undernutrition based on the CIAF is divided in to (“anthropometric failure” coded as 1) and no failure (“normal” coded as 0). The categories are grouped in to: A) no failure (normal); B) wasting only; C) wasting and underweight; D) wasting, underweight and stunting; E) underweight and wasting; F) stunting only; Y) underweight only (Table 1). The anthropometric failure is the total amount of undernutrition or sum of category of wasting only (B); wasting and underweight (C); wasting, underweight and stunting (D); underweight and wasting (E); stunting only (F); underweight only(Y). At the same time, the CIAF index can be used to detect some anthropometric failures [32].

**Table 1:** Category of anthropometric failure in under five children using Composite Index of Anthropometric (CIAF)

Group	CIAF	Description of the level	Wasting	Stunting	Underweight
A	No failure	Normal WAZ, HAZ and WHZ	No	No	No
B	Wasting only	WAZ < -2SD, bun normal HAZ and WHZ	Yes	No	No
C	Wasting and underweight	WAZ and WHZ < -2SD, but HAZ normal	Yes	No	Yes
D	Wasting, underweight and stunting	WAZ, WHZ and HAZ < -2SD	Yes	Yes	Yes
E	Stunting and underweight	HAZ and WHZ < -2SD, but WAZ normal	No	Yes	Yes
F	Stunting only	HAZ < -2SD, but normal WAZ and WHZ	No	Yes	No
Y	Underweight only	WHZ < -2SD, but normal HAZ and WAZ	No	No	Yes

*Source*: The concept of Composite index of Anthropometric Failure (CIAF) by Kuiti and Bose: Revisited and Revised (2018).

*Region*: Regions (Amhara, Tigray, Oromia, SNNP and Harari) which livelihood mainly based on agriculture classified as agrarian (coded as 2); regions (Afar, Somali, Gambella, and Benishangul-Gumuz) which livelihood mainly based on nomadism classified as pastoralist or emerging regions (coded as 1); and regions or city administrations (Addis Ababa and Dire Dawa) which livelihood mainly based on employment and trade classified as urban (coded as 0) [33, 34].

*Community poverty level*: The household wealth status of the community below the median was considered as poor or greater /equal to the median was considered as a rich community wealth index.

*Community women literacy*: Community considered as literate if at least 50% of women in the community attend at least primary education and illiterate if women in the community had no education or only less than half proportion of women in the community educated.

### Data collection

Ethiopian Public Health Institute (EPHI) recruited and trained 151 health professional field staff for the main fieldwork to serve as female interviewers, female anthropometrics, female computer-assisted personal interview (CAPI) supervisors, field supervisors, regional coordinators, and their respective reserves. A household questionnaire, woman questionnaire and man questionnaire were completed at every selected household from each cluster. Questionnaires captured demographic, socio-economic and household characteristics, child characteristics, and child caring practices, and maternal caring practices data. Information on general demographics of the household was collected from the female head of the household. Child’s age was based on birth, health records available at the household or self-reports of the mother or caretaker using an event calendar. Anthropometric indicator length/height-for-age was determined for under five children using current WHO growth standards [14].

### Anthropometric measurements

The length of children aged < 24 months was measured during the EDHS in a recumbent position to the nearest 0.1 cm using a locally made measuring board (Shorr Board®) with an upright wooden base and moveable headpieces. Children ≥ 24 months were measured while standing upright. The length/height-for-age Z-score, an indicator of nutritional status, was compared with reference data from the WHO Multicenter Growth Reference Study Group, 2006. Children whose height-for-age Z-score is < -2 SD from the median of the WHO reference population are considered stunted (short for their age) [14].

*Weight*: Weight measurement was taken after children were undressed (no shoes, dresses and wet hat). For a child who stands on the weighing scale calmly, the measurement was taken in the nearest 0.1 kg. In the time of refuse to be scaled, children’s mother carried and stood on the scale. Finally, the child actual weight was registered by subtracting mother’s weight from mother and child weight [14].

### Quality assurance

The pretest for the 2019 EDHS was performed. EPHI recruited and trained the main field work to serve as team supervisors, field editors, interviewers, secondary editors, and reserve interviewers. In addition, individuals were recruited and trained on how to collect biomarker data, including taking height and weight measurements, testing for anemia by measuring hemoglobin levels.

### Data processing and data analysis

The data management was done through STATA/MP 17.0 statistical analysis software package. The full data set was down loaded from MEASURE DHS website. Some continuous variables were recoded to categorical variables. Data cleaning was performed before any statistical analysis. The kids recode (KR) data set in STATA file is the data set containing the outcome and predictor variables of this study. The data was explored in different ways. The “SVY set” command was used for considering complex survey design. The “iweigh” and “pweight” commands were used for descriptive statistics and regression model respectively for sampling weigh adjustment.

This study was based on secondary data analysis of 2019 EDHS by adjusting sample weights. Categorical characteristics and outcome of the study was described in terms of percentage and frequencies. Tables, bar graph and pie chart were used to present the data for some selected variables which had significant association with CIAF. A bi-variable multi-level logistic regression analysis was carried out to see the crude effect of each independent variable on CIAF, and then variables with *p*. value of < 0.2 were entered to the multivariable multi-level binary logistic regression model. The subject matter knowledge, clinical and social significance, and evidence from the literature also considered for candidate variable selection.

The prevalence of CIAF was estimated with 95% of confidence interval. Summary statistic (mean, median, SD, and IQR) and AOR with 95% confidence intervals were estimated at 0.05 level of significance to identify important predictor variables of CIAF.

Intra class correlation (ICC); median odds ratio (MOR) and proportional change in variance (PCV) statistic were calculated to measure the variation between clusters (the random effect variable). The deviance information criterion (DIC) statistic was calculated for the different models (individual level, community level and both individual and community level) fitted with logit, probit and cloglog link functions. The DIC was used to evaluate and compare model performance of the full model and the reduced model. A model with lower DIC was considered as one with a better fit.

## Results

### Socio-demographic characteristics of respondents

A total of 5,530 under five children (weighted samples) were included in this study. From this study finding 2,521 (50.90%) children were males. Three thousand seven hundred twelve (74.06%) of the children was residing in rural area of Ethiopia. Two thousand nine hundred sixty two (59.82%) of the children were from 24–59 months, while 1469 (29.66%) of the children were 6–23 months. Three thousand seventy one (79.13%) of the children had short preceding birth interval (less than 24 months). Two thousand six hundred fifty four (53.59) of the children were from mothers who have no formal education. The median age of the children’s mothers were 28 years old (IQR = 8). 4755 (96.02%) of the mothers were married (Table 2).

**Table 2:** Frequency and percentage distribution of study characteristics

Variables	Categories	Count	Percent (%)
Age of child	< 6 months	521	10.52
	6–23 months	1469	29.66
	24–59 months	2962	59.82
Birth order	1st child	1060	21.41
	2nd or 3rd child	1599	32.29
	4th or above	2293	46.30
Type of birth	Singleton	4844	97.82
	Multiple	108	2.18
Educational level of the mother	Secondary education and above	546	11.02
	Primary education	1752	35.39
	No formal education	2654	53.59
Age of the mother in years	15–24	1134	22.90
	25–34	2674	54.00
	35–49	1144	23.10
Religion of the mother	Orthodox	1697	34.28
	Protestant	1362	27.50
	Muslim	1796	36.27
	Others	97	1.95
Current marital status of the mother	Married	4755	96.02
	Single	18	0.36
	Divorced	121	2.45
	Widowed	58	1.17
Place of delivery	Home	2,518	50.89
	Health facility	2,429	49.11
Parity	Primi parity	788	15.92
	Multi party	1,785	36.08
	Grand multi parity	2,375	48.00
Number of under five children in the household	1 child	1960	39.59
	2 children	2301	46.47
	3–5 children	690	13.94
Number of household members	1–4 family members	1,422	28.74
	5–9 family members	3,172.2926	64.12
	≥ 10 family members	353.152023	7.14
Wealth index of the household	Richest	918	18.53
	Rich	874	17.64
	Middle	930	18.79
	Poor	1093	22.07
	Poorest	1137	22.96
Source of drinking water	Improved	3160	63.80
	Unimproved	1792	53.59
Type of toilet facility	Improved	786	15.88
	Unimproved	4166	84.12
Community women literacy	Illiterate	2,430	49.11
	Literate	2,518	50.89
Community poverty level	Low	2,741	55.40
	High	2,207	44.60
Administrative region of Ethiopia	Urban based	164	3.31
	Agriculture based	4295	86.74
	Pastoralist	493	9.95

### Prevalence of composite index of anthropometric failure (CIAF) and subgroups of CIAF in under five children in Ethiopia

**Fig. 1:**
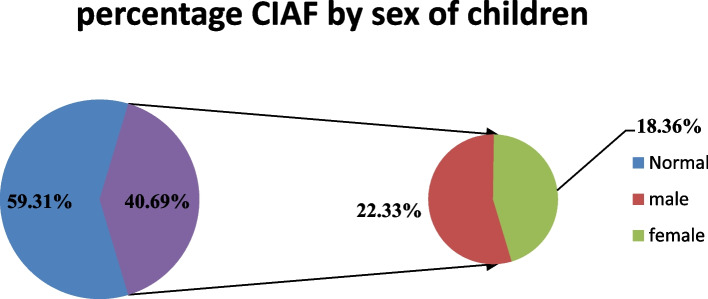
Composite index of anthropometric failure by sex

**Fig. 2:**
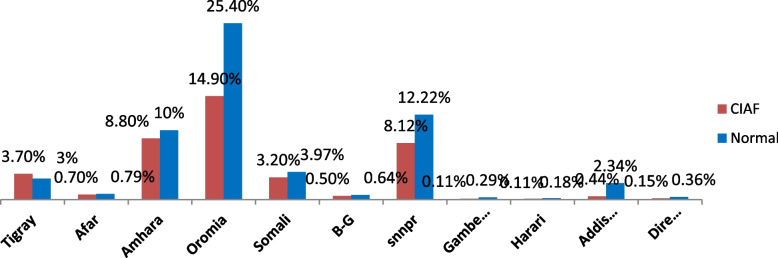
Composite index of anthropometric failure by Ethiopian administrative regions

In this study the prevalence of CIAF was 40.69% (95% CI: 39.41%, 42.00%) in Ethiopia in 2019 which is the latest national data ( \* MERGEFORMAT Fig. 1). From this, the highest share of CIAF was from Oromia region (14.92%) followed by Amhara region (8.78%) and Southern Nations nationalities & people region (8.12%) in Ethiopia. While the lowest share was from Gambella region (0.11%) and Harari region (0.11%) ( \* MERGEFORMAT Fig. 2).

**Fig. 3:**
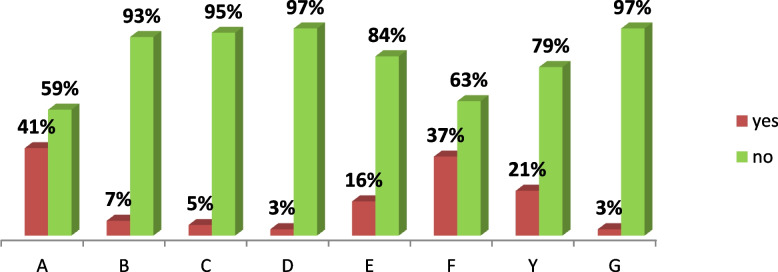
Subgroups of composite index of anthropometric failure

From the subgroups of anthropometric failures in under five children; subgroup F (stunting only) was the highest (37%), subgroup Y (underweight only) was the second (21%), and subgroup B (wasting only) was the third (7%). The top least were subgroup D (stunting and underweight and wasting) (3%) and subgroup G (stunting and wasting) (3%) ( \* MERGEFORMAT Fig. 3).

### Predictors of composite index of anthropometric failure in under five children in Ethiopia

#### Random effect analysis and model fit statistics

All of the random effect analysis parameters favor the final model (model IV) as the best fit model. From the intra class correlation coefficient estimate indicates evidence of substantial clustering which is the proportion of the total variation attributable to variation between clusters, or as correlation between under five children with in the same cluster. The ICC in the model I (null model) indicates that about 11.79% (95% CI: 8.99%, 15.32%) of the variability in composite index of anthropometric failure (CIAF) was attributed to the community/cluster/enumeration area level variability. The MOR in the model I also revealed that if two under five children are taken from two different clusters i.e. one from a cluster with higher CIAF and one from a cluster with lower CIAF) the odds of having CIAF among who came from cluster with higher CIAF was 1.88 times higher as compared to their counter parts. Moreover, the PCV in the final model (model IV) showed that about 44.02% of the variability in the CIAF was explained by both community level and individual level factors. Regarding model fitness, model IV (full model) was the best fitted model since it had the lowest deviance and this model was used to assess predictors of CIAF (Table 3).

**Table 3:** Random effect analysis and model fit statistics for predictors of composite index of anthropometric failure among under five children in Ethiopia

Parameter	Model I	Model II	Model III	Model IV
Community level variance	0.440	0.268	0.242	0.246
ICC	0.1179	0.0754	0.0686	0.0696
MOR	1.88	1.64	1.600	1.602
PCV	Reference	0.3903	0.4492	0.4402
Log likelihood	-3360.3141	-2454.1886	-3234.3828	-2447.9678
Deviance	6720.6282	4908.3772	6468.7656	4895.9356

### Fixed effect analysis

Bi-variable analysis was performed to identify potential candidate variables for the multivariable analysis using 0.2 level of significance. Multivariable analysis was performed to identify significant predictor variables of composite index of anthropometric failure (CIAF) among under five children in Ethiopia. Individual level (level 1) predictor variables and community level (level 2) predictor variables were assessed in model II and model III. Finally, model IV (full model) was fitted to identify important significant predictor variables of CIAF.

In the multivariable analysis, the odds of having CIAF was lower among females 0.71 (AOR: 0.71; 95% CI: 0.56, 0.89) as compared to males keeping the other covariates constant. The odds of having CIAF was higher among under five older age groups 6–23 months 1.64 (AOR: 1.64; 95% CI: 1.10, 2.44), 24–59 months 2.85 (AOR: 2.85; 95% CI: 1.85, 4.38) as compared with those whose ages were below 6 months. Children belonging to mothers with primary education, and no formal education was 1.80 (AOR: 1.80; 95% CI: 1.02, 3.17) and 2.15 (AOR: 2.15; 95% CI: 1.19, 3.89) respectively times higher odds of CIAF as compared to those who had secondary or higher education. Under five children from middle households and poorest households had 2.08 (AOR: 2.08; 95% CI: 1.12, 3.86) and 2.44 (AOR: 2.44; 95% CI: 1.22, 4.88) respectively times higher odds of CIAF as compared to those who were from richest households. Preceding birth interval less than 24 months had 1.27 (AOR: 1.27; 95% CI: 1.02, 1.58) times CIAF as compared to those who had greater than or equal to 24 months. In protestant religion followers the odds of CIAF was 0.69 (AOR: 0.69; 95% CI: 0.52, 0.92) times higher as compared to orthodox followers. Children belonging to divorced mothers had 0.47 (AOR: 0.47; 95% CI: 0.56, 0.92) times odds of CIAF as compared its counterpart married mothers. Regarding to the community level variables; children from high community women literacy had 0.74 (AOR: 0.74; 95% CI: 0.56, 0.98) times higher as it is compared to its counterpart. Regarding the Ethiopian administrative region, under five children from agriculturally based region had 1.89 (AOR: 1.89; 95% CI: 1.16, 3.07) times higher odds of CIAF as compared to under five children from the urban based region (Table 4).

**Table 4:** Predictors of composite index of anthropometric failure among under five children in Ethiopia

Variable	Category	Model I	Model II	Model III	Model IV
Sex of child	Male		1.00		1.00
	Female		0.71 (0.56, 0.89)		0.71 (0.56, 0.89)*
Age of child	< 6 months		1.00		1.00
	6–23 months		1.63 (1.10, 2.43)		1.64 (1.10, 2.44)*
	24–59 months		2.84(1.84, 4.37)		2.85(1.85, 4.38)*
Type of birth	Single		1.00		1.00
	Multiple		2.24(0.94, 5.38)		2.25(0.95, 5.33)
Birth interval	≥ 24 months		1.00		1.00
	< 24 months		1.27(1.02, 1.58)		1.27(1.02, 1.58)*
Education level of the mother	Secondary education and above		1.00		1.00
	Primary education		1.93(1.10, 3.37)		1.80(1.02, 3.17)*
	No formal education		2.37(1.32, 4.27)		2.15(1.19, 3.89)*
Religion of the mother	Orthodox		1.00		1.00
	Protestant		0.65(0.49, 0.86)		0.69(0.52, 0.92)
	Muslim		0.74(0.56, 0.99)		0.78(0.56 1.10)
	Others		0.39(0.21, 0.74)		0.41(0.21, 0.80)
Current marital status of the mother	Married		1.00		1.00
	Single		1.64(0.14, 19.36)		1.53(0.14, 17.41)
	Divorced		0.46(0.22, 0.96)		0.47(0.22, 0.97)
	Widowed		0.81(0.22, 2.97)		0.85(0.23, 3.14)
Place of delivery	Home		1.00		1.00
	Health facility		1.00(0.78, 1.28)		1.03(0.80, 1.32)
Parity	Primi parity		1.00		1.00
	Multi parity		0.86(0.24, 3.06)		0.86(0.24, 3.02)
	Grand multi parity		0.69(.18, 2.69)		0.69(0.18, 2.66)
Number of under five	1 child		1.00		1.00
	2–3 children		1.21(0.91, 0.63)		1.21(0.90, 1.63)
	3–5 children		1.22(0.79, 1.88)		1.22(0.79, 1.88)
Number of household members	1–4 family members		1.00		1.00
	5–9 family members		1.06(0.74, 1.51)		1.06(0.74, 1.51)
	≥ 10 family members		1.06(0.63, 1.80)		1.05(0.62, 1.79)
Wealth index of the household	Richest		1.00		1.00
	Rich		2.03(1.25, 3.31)		1.77 (1.00, 3.15)*
	Middle		2.44(1.50, 3.97)		2.08 (1.12, 3.86)*
	Poor		2.10(1.28, 3.45)		1.74 (0.89, 3.42)*
	Poorest		2.92(1.71, 4.97)		2.44 (1.22, 4.88)*
Type of toilet facility	Improved		1.00		1.00
	Unimproved		0.79(0.55, 1.12)		0.75(0.52, 1.09)
Community women literacy	Illiterate			1.00	1.00
	Literate			0.65(0.51, 0.82)	0.74(0.56, 0.98)*
Community poverty level	Low			1.00	1.00
	High			0.79(0.62, 1.02)	0.88(0.65, 1.20)
Type of residence	Urban			1.00	1.00
	Rural			1.40(1.02, 1.92)	0.940(0.56, 1.58)
Administrative region of Ethiopia	Urban based			1.00	1.00
	Agriculture based			2.27(1.58, 3.27)	1.89(1.16, 3.07)*
	Pastoralist			2.12(1.41, 3.18)	1.480(0.84, 2.60)

NB: Model I is the null model; model II is the model with level 1 predictors; Model III is with level 2 predictors and Model IV is both level 1 & 2 predictor variables (Full model). 1 is reference and * is *p*-value less than 0.05(significant)

## Discussion

These results demonstrate that when conventional indices (stunting, wasting, and underweight) are used alone, they miss a significant number of under-five children who already have multiple anthropometric deficits. This is because conventional indices underestimate the prevalence of under-nutrition and do not provide the overall prevalence of under-nutrition in children. By using the CIAF aggregate measurements of malnutrition, this problem was avoided [29]. Studies conducted in Ethiopia [35–37] focused on either of the conventional indices may be suitable to inform interventions targeting at the reduction of each of the conventional indices alone, whereby, this study may help address the primary causes of undernutrition in the nation in all of its manifestations.

In Ethiopia, composite index of anthropometric failure (CIAF) still remains public health problem. Nationally, the prevalence of CIAF was high (nearly 41%). The finding is below to study findings previously done in Ethiopia (61.30% in 2000, 56.57% in 2005, 51.58% in 2011 and 46.58% in 2016) [38]. The figure is also lower than to the finding in southwest Ethiopia (50.80%)[30]. But similar to study finding in the rural area of the Bogor District in Indonesia (42.12%) [13]. However, the current study higher than those found in Tanzania (38.2%) [22] and various parts of India, including Parwano and Himacha Pradesh (31.9%) [39], rural areas of west Bengal (32.7%)[40]. When compared with the prevalence of under-nutrition among Argentineans (15.1%) [41] and Bangladesh 11.3%[42], in the current research, the prevalence of malnutrition was much higher. The variation with other previous studies may be due to the difference in socio-economic and socio-cultural characteristics of respondents between countries. And the study period is also matters the difference in the findings of different studies.

The findings showed that female children had a lower risk of having CIAF than male children from a similar socioeconomic background. This study is consistent with previous studies [38, 43], It could possibly be a factor in CIAF because of the biological growth and vulnerability of men and the fact that the percentage of male preterm births is higher than that of female preterm births [44].

The results showed that children in the older age group were more likely than those in the younger age group to have CIAF. This is in line with research done in other nations, such as Tanzania and Yemen [15, 22]. This may result from a child being fed a more balanced and nutrient-rich food when they are younger, but as a child gets older, breastfeeding may stop and their body's need for nutrients may rise.

Furthermore, a birth interval of less than 24 months raises the likelihood of being CIAF. The results of this investigation are in line with those of other investigations [45–47]. Individuals with short inter-birth intervals may experience negative effects on their children's nutrition as it might compromise the child's intrauterine growth and quality of care [48].

It was discovered that undernutrition in children was substantially correlated with the mother's educational attainment. Additionally, this conclusion is in line with earlier research showing that maternal education reduces childhood undernutrition [38, 42, 49]. One explanation could be that moms' formal education provides them with knowledge that enables them to practice healthy eating habits and other related behaviours that avoid undernutrition. Additionally, compared to mothers who lack education, educated mothers are more likely to seek medical attention for childhood ailments [50]. Greater use of health care, adoption of contemporary medical procedures, and more female autonomy are all correlated with better maternal education. These factors then impact decisions about health that enhance the nutritional outcomes for children [51].

According to the current study, children from lower-class families are more likely than their counterparts to be impacted by the CIAF. This is consistent with earlier research done in many nations [42, 43, 52]. This could be the case because the wealthiest households can afford to buy different types and amounts of food for their kids, while poorer homes may have less access to health care services than wealthier ones.

According to this study, the region of Ethiopia significantly affects the CIAF of children. Compared to children living in cities, children from rural backgrounds are more likely to have CIAF. A plausible rationale for this could be because youngsters residing in metropolitan areas have superior living standards and quicker access to sustenance [53]. Furthermore, the current research shows that children from high community women literacy had less likely to have CIAF than their counterparts from low community women literacy. This could be due to educated community women are more likely to follow basic nutrition and hygiene practices. Additionally, compared to their counterparts from low community women literacy, children from high community women literacy had a lower likelihood of having CIAF. This may be because community women with higher levels of education are more likely to adhere to basic cleanliness and dietary guidelines, which may lower the risk of anthropometric failure. Another reason could be that informed community women are aware of the importance of nutrition and are able to prevent undernutrition by understanding the information offered by the media or medical professionals.

## Conclusions

This study found that composite index of anthropometric failure (CIAF) was high in Ethiopia. Factors both at the individual level and at the community level were predictors of composite index of anthropometric failure. Individual level predictors like age of child, sex of child, preceding birth interval, mother’s education, household wealth index were identified as important predictors of CIAF of the under five children. Whereas community level predictors such as community women literacy and administrative regions of Ethiopia were identified as predictors of CIAF in under five children.

Therefore, giving special attention to male children, older age of children, those children from mothers’ who had no formal education, and those who are from poor socioeconomic to decrease the burden of composite index of anthropometric failure in under five children in Ethiopia. Besides, increasing the community women literacy can decrease the CIAF in under five children. Attention is also should be given for agricultural based administrative regions of Ethiopia.

## Data Availability

The dataset used during the current study is available from the corresponding author upon reasonable request.

## Abbreviations

BMI: Body Mass Index
BDHS: Bangladesh Demographic and Health Survey
CAPI: Computer-Assisted Personal Interview
CIAF: Composite Index of Anthropometric Failure
DIC: Deviance Information Criterion
EDHS: Ethiopian Demographic and Health Survey
EFMoH: Ethiopian Federal Ministry of Health
EMDHS: Ethiopian Mini Demographic and Health Survey
EPHI: Ethiopian Public Health Institute
GBD: Global Burden of Diseases
ICC: Intra Class Correlation
LMICs: Low- and Middle-Income Countries
MOR: Median Odds Ratio
PCV: Proportional Change in Variance
SDG: Sustainable Development Goal
SSA: Sub-Saharan Africa
UNICEF's: United Nations Children's Fund
WHO: World Health Organization
